# Supplementary material for: Effects of indoor air pollution due to solid fuel combustion on physical growth of children under 5 in Sri Lanka: A descriptive cross sectional study
Source: PLoS One. 2021 May 25;16(5):e0252230. doi: 10.1371/journal.pone.0252230 (PMC8148308; doi:10.1371/journal.pone.0252230)
Supplement: Attachment — Submitted filename: Responses to reviewers.docx [file pone.0252230.s001.docx]

**Responses to reviewer comments**

| **Reviewer comment – Reviewer 01** | **Response** |
| --- | --- |
| Indoor air pollution is strongly spatiotemporally dependent. A child may be hurt more severely by indoor air pollution as the cooking lasts longer even if the pollution is comparable or lighter. Analysis with the absence of exposure time will, therefore, compromise the results. | We agree with the reviewer’s comment. Exposure time was assessed and there was only one child from the low exposure group and 2 children from high exposure group who spent time near stove more than one hour during the exposure measurement. Furthered the maximum time spent near the stove per day by a child was 2 hours (one child)(revised in the manuscript line 282-283).  As suggested by the reviewer, indoor air pollution is spatiotemporally dependent. We were only able to measure exposure for a two hour period during the cooking of lunch, the main meal of the family. We have mentioned this as a limitation (line numbers 354 to 358 in revised manuscript). |
| In the high-exposure group, unprocessed biomass, which includes wood, grasses, crop residues or animal dung, was used for cooking. Coal and kerosene were also used. In the low-exposure group, they used liquefied petroleum gas or electricity, with the former producing particles, SO2, NOx, CO, non-methane total hydrocarbons (incomplete combustion), etc., at a lesser extent. Since different fuel combustion produces different air pollutants, which may exert different harmful effects on health, the authors need to present in detail the situation of households using different fuels. Besides, they should pay attention to the history of fuels used, because the type of fuels might be changed during the study period. | We monitored the use of different fuels during the study. The mothers of these children were recruited during their pregnancy and continuously followed. Since, recruitment of these mothers there was no change in the principal cooking fuel. 100% of the low exposure group households were using liquid petroleum gas for cooking as the primary source of fuel. In the high exposure group households, 94% of the households were using firewood as the primary cooking fuel. As we present baseline results of the study on children under 5, any change in fuel is unlikely to have affected the results.  (Revised in the manuscript line 191-195) |
| Air quality was measured in 115 out of 242 households. Why were the measurements not taken from all households? Similarly, analysis was performed based on data from the subgroups (e.g., Table 7). Comparisons conducted with smaller sample sizes would potentially bias the results. | Due to the limited availability of resources, we measured the air quality levels in a subsample of the cohort. That was a limitation of the research and included in the limitation section. (Revised in the manuscript line 353-355) |
| Inclusion and exclusion criteria were not presented. Was the child with premature birth, obesity or other abnormal conditions (prenatally and postnatally) included? Are there any twins? | Inclusion and exclusion criteria have been included.(Revised in the manuscript line 99-103) |
| Description for data analysis is not sufficient. How to compare the anthropometric parameters, e.g., values of weight-for-age z-scores expressed as mean and SD (Table 03), between the two groups? There is a lack of description regarding confounding factors in Method session. The authors should give the reason why they chose exposure status, age, sex, monthly family income and parental education as the confounders. Multicollinearity should be considered for tightly correlated variables if any. Did they consider the co-effects of outdoor-level air pollution, which may not be negligible especially in developing countries? | Z scores for anthropometric measurements were obtained from WHO Anthro software. Mean and SD was calculated for those values. Comparisons between the high and low exposure groups was done using independent sample t-tests. The anthropometric parameters for different socio-demographic groups were also compared using the independent sample t-test.  The reason why age, sex, monthly family income and parental education were considered were included as potential confounders as they were significant on bivariate analysis (unadjusted). In the multiple regression model, multicollinearity between independent variables would have been considered in the final output.  We did not consider co-effects of outdoor air pollution as we did not measure. We have included it as a limitation. (Revised in the manuscript line 354-355) |
| There are strong relationships of different toxic pollutants to one another with regard to air pollution on health. There are no models of multi-pollutants to reflect the texture of air pollutants. The average concentrations of CO, CO2 and PM2.5 for both groups should be given. What did the authors evaluate the temperature and humidity for? | We were able to measure only CO, CO2 and PM2.5. We have included a statement as a limitation (Revised in the manuscript line 364-365) The air quality measurements were obtained with the temperature and humidity being automatically measured by the testing instrument. As we did not observe any significant variation in those measurements, we did not analyze those data. Therefore, that part was not included in the manuscript. Data presented in the table 06 are related to the sub sample of households (115) where 55% belonged to high exposure group. An additional table has been included (Table 05) |
| The authors may have to evaluate the weakness of the study in the Discussion session. | Included in the revised manuscript giving the limitations as a subsection |
| “Any detrimental effect on growth during early childhood is irreversible” (Line 51): This may not be true because some effects are temporary and reversible. | The meaning of “detrimental effect” defined here is about irreversible effects and the sentence is revised in the manuscript |
| form → from (Line 92). | Revised the error |
| ‘exposed group’ (Line 96) and “control group (Line 97): It is more appropriate to use “high exposure group” and “low exposure group”, as used by the authors in other places. | Corrected in the manuscript |
| Statements such as “… to collect socio-demographic data and the principal source of cooking” (Line 102) should go to the Method session. | It is in the methods section |
| “PM2.5 and Carbon Monoxide (CO) concentrations were measured …” (Line 144-145): It should be PM2.5, CO2 and CO to be measured. | This has been corrected in the revised manuscript. |
| I suggest the sublevel headings be used for the Results session. | Included in the manuscript |
| “Stunting and wasting were …” (Line 200-201) → The prevalence (or ratio) of stunting and wasting was… | Corrected in the manuscript |
| “P” whether in uppercase (as in Table 3) or in lowercase (as in Table 2) should be consistent. | Converted to lowercase in table 3. |
| Table 1 (Page 9): “Family income”: Is it monthly income? (indicated in Line 242) | Corrected in tables 1 and 3. |
| “Entire study population” column in Table 2 (Page 10): “n = 1.7”? | It was a typing error. Corrected in the revised manuscript |
| Line 213 and 214: “vs” → “vs.”. | Corrected in the manuscript |
| Line 235: “… to children three …”→ “… to children of three …”; “(Tables 4 and 6)” → (Tables 4-6). | Corrected in the manuscript |
| Line 306-308: Lacking reference(s). | Reference given |
| “the findings of a study amongst older children may be different”: No supportive data. | It was mentioned in the discussion where it needs a follow up for a longer time and that has not been assessed in this study. (Revised in the manuscript line 317) |
| “However, as households with higher IAP … , IAP would contribute … on physical growth” (Line 327-330): The authors give insufficient rationales that IAP contributed significantly because IAP is not definitely correlated with other adverse conditions. | In this section, it was meant to address the multifactorial effects of growth which was addressed in this study and not completely addressed in some aspects such as nutrition. Therefore, we discussed the cumulative effect which can be a reinforcing factor for growth faltering. |
| Line 336: “mg/m3” → μg/m3 | Corrected in the manuscript |
| “respirable particulate matter” (Line 341): Do they refer to “inhalable particulate matter”? If so, “PM2.5” that follows can be omitted. | Corrected in the manuscript |
| I suggest the name of country be added in the title and deleted in “Key Words”. | Corrected in the manuscript |
| Line 371-373: Since it is not necessary to repeat the funding, the Acknowledgements session can be deleted. | Corrected in the manuscript |
| **Reviewer comment – Reviewer 02** | **Response** |
| As this is an observational study, the authors may wish to change the title to reflect that appropriately. | Corrected in the manuscript |
| The supplementary information is another article on the effect of indoor air pollution on childhood respiratory diseases published by the authors. Is this intentional or a mistake? | It was given intentionally as this is the same study population and the same sociodemographic data are repeated in both. |
| The choice of covariates in the regression is not clear to me. Why did the authors not use all the covariates in the regression analysis? | We have now explained it under data analysis. Only the significant covariates on bivariate analyses were included in the multiple linear regression analyses.(Revised in the manuscript line 170-173) |
| The results in tables 4-6 can probably be presented more succinctly (in one table). | Corrected in the manuscript |
| Did the authors run regressions with the quantitative measures of air pollutants as the independent variable(s) rather than exposure group? I would be interested in seeing the results for these regressions as well. | We did not do this analysis as air pollutant levels were measured in only 114 households. |
| The manuscript does not interpret the regression estimates comprehensively (sign, significance and size). The size of the effect should probably be described in the results section. | This has been done in the revised manuscript. |
| In the discussion, the authors refer to the results of the correlation between severe anthropometric failure and exposure group after discussing the results of the regressions. If the authors wish to establish the (absence of a) relationship between severe anthropometric failure and exposure group, they might want to run regressions with severe stunting, severe wasting, and severe underweight as the dependent variable. | We did not do this analysis as the numbers with severe forms of malnutrition were too few to perform any analysis. |
| The limitations of the study should be described in the discussion. | Added as a separate section. (Revised in the manuscript line 353-365) |
